# Analysis of Non-Relapsed and Relapsed Adult Type Granulosa Cell Tumors Suggests Stable Transcriptomes during Tumor Progression

**DOI:** 10.3390/cimb44020048

**Published:** 2022-01-28

**Authors:** Noora Andersson, Ulla-Maija Haltia, Anniina Färkkilä, Swee Chong Wong, Katja Eloranta, David B. Wilson, Leila Unkila-Kallio, Marjut Pihlajoki, Antti Kyrönlahti, Markku Heikinheimo

**Affiliations:** 1 HUSLAB, Helsinki University Hospital, Haartmaninkatu 4, 00290 Helsinki, Finland; noora.andersson@hus.fi; 2 Pediatric Research Center, Children’s Hospital, University of Helsinki and Helsinki University Hospital, Tukholmankatu 8, 00290 Helsinki, Finland; katja.eloranta@helsinki.fi (K.E.); antti.kyronlahti@helsinki.fi (A.K.); markku.heikinheimo@helsinki.fi (M.H.); 3 Department of Obstetrics and Gynecology, University of Helsinki and Helsinki University Hospital, Haartmaninkatu 2, 00290 Helsinki, Finland; ulla-maija.haltia@helsinki.fi (U.-M.H.); anniina.farkkila@helsinki.fi (A.F.); leila.unkila-kallio@helsinki.fi (L.U.-K.); 4 Research Program for Systems Oncology, University of Helsinki and Helsinki University Hospital, Haartmaninkatu 8, 00290 Helsinki, Finland; 5 MediSapiens Ltd., Vuorikatu 14 B, 00100 Helsinki, Finland; sweechong.wong@gmail.com; 6 Department of Pediatrics, Washington University in St. Louis, 660 S Euclid Ave, St. Louis, MO 63110, USA; wilson_d@wustl.edu; 7 Department of Developmental Biology, Washington University School of Medicine, 660 S. Euclid Avenue Campus Box 8103, St. Louis, MO 63110, USA

**Keywords:** adult-type granulosa cell tumor, archival FFPE samples, transcriptomic profiling, tumor heterogeneity, tumor evolution

## Abstract

Adult-type granulosa cell tumor (AGCT) is a rare ovarian malignancy characterized by slow growth and hormonal activity. The prognosis of AGCT is generally favorable, but one-third of patients with low-stage disease experience a late relapse, and over half of them die of AGCT. To identify markers that would distinguish patients at risk for relapse, we performed Lexogen QuantSeq 3′ mRNA sequencing on formalin-fixed paraffin-embedded, archival AGCT tissue samples tested positive for the pathognomonic Forkhead Box L2 (*FOXL2*) mutation. We compared the transcriptomic profiles of 14 non-relapsed archival primary AGCTs (follow-up time 17–26 years after diagnosis) with 13 relapsed primary AGCTs (follow-up time 1.7–18 years) and eight relapsed tumors (follow-up time 2.8–18.9 years). Non-relapsed and relapsed primary AGCTs had similar transcriptomic profiles. In relapsed tumors three genes were differentially expressed: plasmalemma vesicle associated protein (*PLVAP*) was upregulated (*p* = 0.01), whereas argininosuccinate synthase 1 (*ASS1*) (*p* = 0.01) and perilipin 4 (*PLIN4*) (*p* = 0.02) were downregulated. *PLVAP* upregulation was validated using tissue microarray RNA in situ hybridization. In our patient cohort with extremely long follow-up, we observed similar gene expression patterns in both primary AGCT groups, suggesting that relapse is not driven by transcriptomic changes. These results reinforce earlier findings that molecular markers do not predict AGCT behavior or risk of relapse.

## 1. Introduction

Adult-type granulosa cell tumor (AGCT) is a rare sex-cord stromal tumor that accounts for 5% of ovarian malignancies [1]. AGCT is characterized by its slow growth pattern and propensity for late relapse. AGCTs are typically hormonally active and the majority of them produce estrogen, which leads to endometrial proliferation and symptomatic uterine bleeding. Consequently 80–90% of the tumors are diagnosed at an early stage [2]. However, serum estradiol level is not a reliable tumor marker since 30% of cases do not secrete estrogen due the lack of theca cells in tumor stroma [3]. AGCTs also secrete inhibin A and B, inhibin B being more frequently elevated [3]. A somatic missense mutation in Forkhead Box L2 (*FOXL2*) (c.402C > G; C134W) is present in 95–97% of AGCTs and is considered pathognomonic [4,5]. This mutation can be used as a sensitive and specific test for molecular differential diagnosis of AGCT [6].

The prognosis of AGCT is generally favorable with 5- and 10-year overall survival rates of 98% and 84%, respectively, in *FOXL2*-validated cohorts [5]. Nevertheless, up to one-third of AGCTs relapse [7,8], and 50–80% of the relapsed patients die of the disease [8,9]. Median relapse time is 7.2 years in histologically and molecularly defined AGCT cohorts [5]. Tumor rupture is the strongest predictive factor for relapse [10].

Recently, whole-genome and whole-exome sequence analyses of fresh-frozen pathological specimens have identified recurring telomerase reverse transcriptase (*TERT*) promoter (−124C > T) and lysine methyltransferase 2D (*KMT2D*) truncating mutations in AGCTs. These molecular variants were associated with more aggressive disease [11,12,13]. Currently, however, there are no molecular markers to identify patients with increased risk of relapse or progression [12].

In this study we compared the transcriptomic profiles of archival primary and relapsed AGCTs to identify markers that would distinguish patients with risk of tumor relapse in AGCT patients. We analyzed formalin-fixed paraffin-embedded (FFPE) tissue samples from a cohort of patients with comprehensive clinical data and follow-up time up to 33.9 years. We profiled the gene expression pattern of these samples using 3′ mRNA sequencing.

## 2. Materials and Methods

### 2.1. Patient Samples

**Figure 1:**
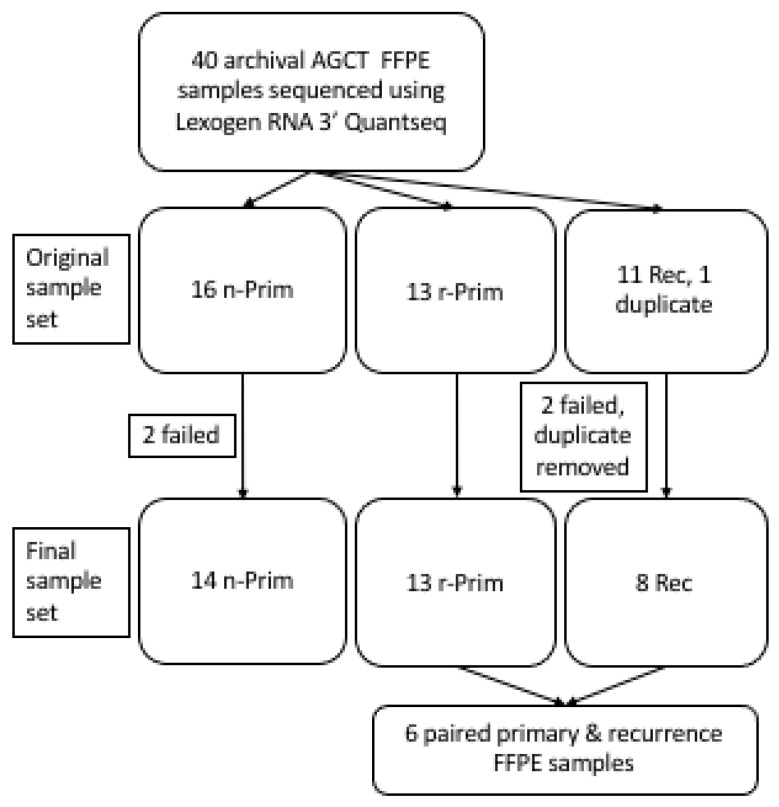
A cohort of archival AGCT samples was sequenced using Lexogen RNA 3′ QuantSeq. Originally 40 FOXL2 mutation validated AGCT samples were collected for this study.

The AGCT samples analyzed in this study were tested positive for the *FOXL2* (c.402C > G, p.C134W) mutation [14] and are summarized in Figure 1. Originally, 40 FFPE samples were included into the study. Two of the FFPE samples failed library preparation (one non-relapsed primary (n-Prim) and one relapse (Rec)) and two samples were excluded from analyses due to poor sequencing read counts (one n-Prim and one Rec). One duplicate sample was also excluded from the analysis (Rec). The final study cohort included 35 archival FFPE AGCT samples from 29 patients: 14 primary AGCT samples from patients who did not have a relapse in 17 to 26 years (median 19.1 years) of follow-up (n-Prim, Group 1), 13 primary AGCT samples from patients who had a relapse in 1.7 to 18 years (median 8.5 years) after primary tumor diagnosis/surgery (r-Prim, Group 2), and 8 from relapsed tumors that relapsed in 2.8–18.9 years (median 6.0 years) from original diagnosis (Rec, Group 3), including 6 patients with primary-relapse tumor pairs (Figure 1). The archival age of the FFPE samples ranged from 4 to 43 years altogether, with a median of 20 years (Table 1). RNA extracted from FFPE samples was partly degraded; RNA integrity number (RIN) of the samples ranged from 1.1 to 2.9 (median RIN 2.5). Clinical and molecular characteristics of the AGCT patients in each group are presented in Table 1. *TERT* promoter mutation status of 23 samples out of the 35 was previously characterized in Pilsworth et al. [11]. To validate the FFPE results, six fresh frozen tumor samples (three primary and three relapsed tumors that relapsed in 1.0–22.6 years after primary tumor diagnosis/surgery) were included to the study. According to Finnish legislation, no written or verbal consent was needed from the patients for using the archival tissue samples coupled with clinical data. All experiments were carried out in accordance with applicable regulations and ethical guidelines. The ethics committee of Helsinki University Central Hospital and the National Supervisory Authority for Welfare and Health in Finland approved the experimental protocols used in this study.

### 2.2. RNA Extraction and RNA Quantity and Quality Assessment

Ten AGCT FFPE tissue sections of 5-µm thickness were mounted on objective slides, and areas with >80% AGCT cellularity (based on hematoxylin and eosin slide review) were scraped off into a microcentrifuge tube. Total RNA was extracted from the FFPE tissue using Maxwell RSC RNA FFPE kit (Promega, Madison, WI, USA), according to instructions. Quality and quantity of the extracted RNA samples were assessed with a 2100 Bioanalyzer using RNA 6000 Pico Kit (Agilent, Santa Clara, CA, USA) and Qubit RNA BR kit (Thermo Fisher Scientific, Waltham, MA, USA). For genomic DNA contamination measurement, a Qubit DNA BR kit (Thermo Fisher Scientific, Waltham, MA, USA) was used. In order to avoid the batch effect, all RNA samples were extracted using the same automated extraction system by one researcher. Furthermore, all samples were sequenced on the same run.

### 2.3. Library Preparation and RNA Sequencing

Single-indexed mRNA libraries were prepared from 100 ng of RNA with QuantSeq 3′ mRNA-Seq Library Prep Kit FWD (Lexogen GmbH, Vienna, Austria), according to the user guide version 015UG009V0230. ERCC RNA spike-in mix (Life Technologies, Carlsbad, CA, USA) was added as a control to each sample according to manufacturer’s instructions. Quality of libraries was measured using 2100 Bioanalyzer DNA High Sensitivity Kit (Agilent, Santa Clara, CA, USA). Sequencing was performed with HiSeq 2500 System (Illumina, San Diego, CA, USA) in high output run mode using v4 chemistry. Read length for the paired-end run was 2 × 101 bp and target coverage of 5 M reads for each library. QuantSeq 3′ mRNA-Seq Integrated Data Analysis Pipeline on Bluebee^®^ (Lexogen) was used for preliminary quality evaluation of the RNA sequencing data. The data were deposited in the NCBI Gene Expression Omnibus and are accessible through GEO Series accession number GSE190942.

### 2.4. Bioinformatics and Statistical Analysis

To filter out lowly expressed genes, sample read counts were transformed into count-per-million (CPM) values using *edgeR* package [15,16] in R [17]. Genes with more than 0.4 CPM values in at least eight samples were included for further downstream differential expression analyses. After filtering, 21,149 genes were retained. The filtered data were normalized and transformed using the *voom* function from the *limma* package [18], using the normalization factors.

Differential gene expression analyses were conducted in two experiments: the first experiment tested differential gene expression among all three different sample groups (group 1: non-relapsed primary tumors, group 2: relapsed primary tumors, and group 3: relapse tumors), and the second experiment combined group 1 and group 2 to assess differentially expressed genes with Group 3. Differential gene expression analyses were performed using the *lmfit* function also from the *limma* package [18], taking sample age as a covariate. After doing differential expression analysis with limma, multiple testing was accounted for by adjusting the *p*-values based on the false discovery rate (FDR). Cut-off value was set to the Benjamini–Hochberg adjusted *p*-value < 0.05.

### 2.5. RNA In Situ Hybridization

RNA in situ hybridization was performed on a *FOXL2* mutation validated AGCT tissue microarray (TMA) series containing 175 (121 primary and 54 relapsed) tumor samples [13]. This array contains both primary and relapsed tumor samples from 19 different patients (1–5 relapse samples each). We used freshly cut 4.5 μm sections of the AGCT TMA using RNAscope 2.5 HD detection kit-BROWN (#322310, ACDBio, Milano, Italy) for target mRNA detection. In short, tissue sections were baked for 1 h at 60 °C, then deparaffinized and treated with hydrogen peroxide for 10 min at room temperature. Target retrieval was performed for 15 min at 95 °C, followed by protease plus treatment for 15 min at 40 °C. The probes Hs-*PLIN4* (#809051, target region: 2289–3198, ACDBio), Hs-*PLVAP* (#437461, target region: 647–2039, ACDBio), Hs-*ASS1* (#431291, target region: 86–1542, ACDBio), positive control probe Hs-*PPIB* (#313901, target region: 139–989, ACDBio) and negative control probe dapB (#310043, target region: 414–862, ACDBio) were hybridized for 2 h at 40 °C followed by signal amplification steps. The samples were incubated for 45 min with AMP 5–reagent. The sections were next treated with DAB for 10 min at room temperature followed by counterstaining with 50% hematoxylin. The sections were dipped in ammonium water and dehydrated before mounting. Two researchers (N.A. and M.P.) performed the scoring independently and disagreements were resolved by a joint review. The number of dots was classified into four categories from 0 to 3 (0 = negative, 1 = weak, 2 = moderate, 3 = high expression). All TMAs were digitalized using a 3DHISTECH Pannoramic 250 FLASH II digital slide scanner at Genome Biology Unit supported by HiLIFE and the Faculty of Medicine, University of Helsinki, and Biocenter Finland.

## 3. Results

### 3.1. Transcriptional Profiling of Archival AGCT Samples

We performed transcriptional profiling on archival primary AGCT samples without a relapse (n-Prim, *n* = 14), primary AGCTs with relapse (r-Prim, *n* = 13), and relapsed tumors (Rec, *n* = 8), and performed a pairwise comparison between the gene expression profiles of relapsed tumors and their corresponding primary tumors (*n* = 6) (Figure 1).

**Figure 2:**
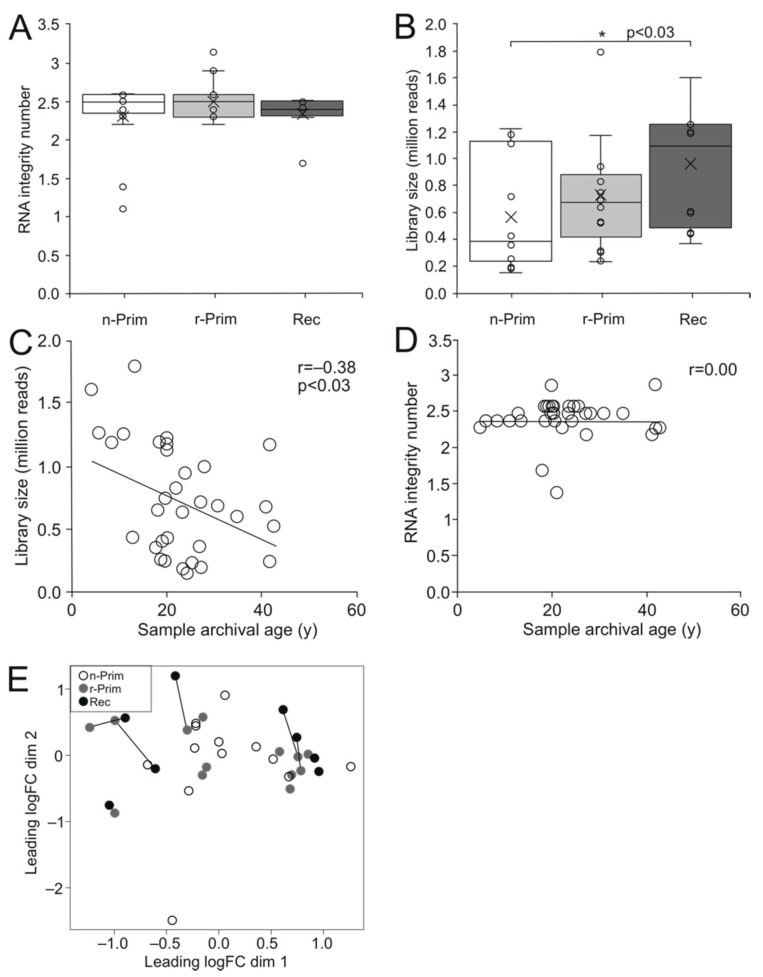
The cDNA library size shows a weak negative correlation with the sample archival age. The RIN values did not differ significantly between the 3 AGCT sample groups (**A**). The cDNA library size was significantly higher in the Rec samples compared to n-Prim tumor samples, *p* < 0.03 (**B**). The cDNA library size had a weak negative correlation with the sample archival age in years (y), r = −0.38, *p* < 0.03 (**C**), but there was no correlation between the RIN value and the sample archival age in years (y) (**D**). We performed a principal component analysis on the sequenced samples to visualize the relationships between the samples. White circle represents the n-Prim, grey circle represents the r-Prim, and black circles represent the Rec samples, respectively (**E**).

Since RNA in FFPE samples is fragmented and partially degraded, we performed gene expression profiling using 3′ mRNA sequencing that is well suited for detecting short transcripts [19]. This sequencing method includes all transcripts that have poly(A) tails, and only one copy of cDNA is generated for each transcript. Therefore, the number of reads directly reflects the number of transcripts of each individual gene, and longer transcripts are not underrepresented in the analysis. We did not find significant difference in RNA integrity among the three sample groups (Figure 2A). The libraries generated from 149,000 to 1,791,263 raw reads (median 651,298) (Figure 2B). The oldest n-Prim samples had significantly fewer reads than the Rec samples (*p* < 0.03) (Figure 2B), and the library size showed a weak negative correlation with sample archival age (r = −0.38, *p* < 0.03) (Figure 2C), while RNA integrity did not (Figure 2D).

We performed a principle component analysis to visualize the distance and relatedness between the three sample populations, and we found that the samples did not cluster into their groups, and that variation in the gene expression pattern was not explained by the sample group (Figure 2E). The distance between the paired samples in the multidimensional scaling plot did not correlate with time to relapse.

### 3.2. Primary AGCT Samples with or without Later Relapse Show Highly Similar Gene Expression Patterns

We next performed differential gene expression analysis to assess global changes in gene expression between the three sample groups. First, we transformed sample read counts into counts-per-million (CPM) values to filter out transcripts expressed at low levels. We included transcripts with more than 0.4 CPM values in at least eight samples for differential gene expression analyses. After filtering, 21,149 transcripts were retained for further analysis. Sample archival age was significantly associated with library size and (*p* < 0.05) in regression analysis (Figure 2C), and we therefore included archival age as a covariate in the subsequent differential gene expression analysis.

We first compared the gene expression patterns between the two primary tumor groups (n-Prim and r-Prim). These two groups showed highly similar gene expression patterns, and no genes with significantly differential expression were identified. To test whether less stringent filtering criteria would uncover more differentially expressed genes between the n-Prim and r-Prim groups, we lowered the read count filtering criteria to >0.3 CPM values in at least 5 samples. With these criteria, the number of transcripts included in the analysis increased by 11% to 23,560, but the two primary tumor groups still did not show significant differential gene expression.

### 3.3. Relapsed AGCT Show Stable Transcriptomic Profiles as Compared to Primary Tumors

**Figure 3:**
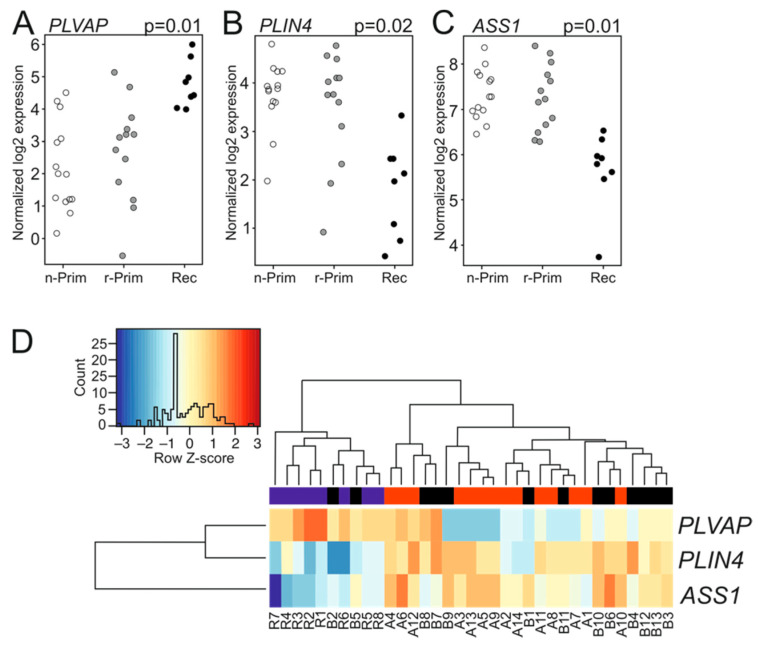
The expression patterns of PLIN4, PLVAP and ASS1 in AGCT samples. The normalized log2 expression values for PLVAP (**A**), PLIN4 (**B**), and ASS1 (**C**) were plotted for each sample and group. White circle represents the n-Prim, grey circle represents the r-Prim, and black circles represent the Rec samples, respectively. Differences in transcript abundance are shown for PLVAP, PLIN4, and ASS1 in AGCT samples (**D**). In the heatmap, red color represents relative increase in abundance; the blue color represents relative decrease, and the white color represents no change. The red color in the bar above the heatmap represents n-Prim, the black color represents the r-Prim, and the blue color represents Rec samples, respectively.

Next, we analyzed the transcriptomic differences between the six primary-relapse tumor pairs. Interestingly, these tumor pairs did not reveal any differentially expressed genes. We then combined both primary tumor groups (n-Prim and r-Prim) and compared their transcriptomic signatures (*n* = 27) to the relapsed tumors (Rec, *n* = 8). Three genes showed differential expression: plasmalemma vesicle associated protein (*PLVAP*) was significantly up-regulated (log FC 2.5, *p* = 0.01) in relapsed tumors (Figure 3A), while perilipin 4 (*PLIN4*) (log FC −2.2, *p* = 0.02) and argininosuccinate synthase 1 (*ASS1*) (logFC −1.6, *p* = 0.01) were significantly down-regulated (Figure 3B,C, respectively). Figure 3D shows the differences in transcript abundance for *PLVAP*, *PLIN4*, and *ASS1* among the three AGCT sample groups. Lowering the read count filtering did not reveal additional differentially expressed genes between the combined primary tumor and relapsed tumor groups. PLVAP is a protein expressed in endothelial cells, and it forms the stomatal and fenestral diaphragms of blood vessels and regulates basal permeability, leukocyte migration, and angiogenesis (reviewed in [20]). PLVAP is upregulated in endothelial cells in several tumors, where it facilitates vascular growth in cancer [20]. *PLIN4* encodes for a perilipin family protein that sequesters lipids by protecting lipid droplets from lipase action, and it may promote chemoresistance of triple negative breast cancer cells [21,22]. *ASS1* is a rate-limiting enzyme in arginine biosynthesis, and its abundance is reduced in various solid tumors, making them auxotrophic for arginine. *ASS1* is highly expressed in gastric cancer and its expression positively correlates with gastric cancer aggressiveness and poor outcome [23]. To validate the FFPE-sample results we sequenced six fresh frozen tumor samples (three primary and three recurrent tumors) along with the FFPE-samples. Differential gene expression analysis did not identify any differentially expressed genes between the primary and recurrent tumors.

### 3.4. Gene Expression Patterns in AGCT Are Independent of TERT Mutation Status

A somatic *TERT* promoter mutation has previously been found in about one-third of AGCT samples [11,12]. We analyzed whether there were differentially expressed genes between *TERT* promoter mutation positive and wild type samples. There were 5 *TERT* mutation positive samples, 3 primary tumors without relapse, and a primary-relapse tumor pair from one patient [11], and 18 cases were wild type for the *TERT* mutation (Table 1). We did not find differentially expressed genes between the *TERT* mutation positive and wild-type samples even with the less stringent filtering criteria.

### 3.5. A TMA Validation Cohort Confirms PLVAP Upregulation Both in Tumor Cells and Endothelial Cells within Relapsed Samples

We next used RNA in situ hybridization to validate the expression patterns of *PLVAP*, *PLIN4,* and *ASS1* in an AGCT TMA containing 121 primary, and 54 relapse *FOXL2* mutation positive AGCT samples. All RNA sequenced samples were included in the TMA array, and it includes 17 matched pairs of primary and relapse tumors from the same patient. The clinical characteristics of the patient samples in this cohort have been described earlier [18]. We used cyclophilin B (*PPIB*) as the control for RNA quality, and samples that were negative for *PPIB* expression were excluded from further analysis. We scored each target probe expression into negative, weak, moderate, or high according to the amount of signal detected, and each target gene expression was normalized to *PPIB* expression, and samples negative for *PPIB* expression were discarded (Table 2). After normalization all positively stained samples were grouped together for correlation analyses (Table 3).

**Figure 4:**
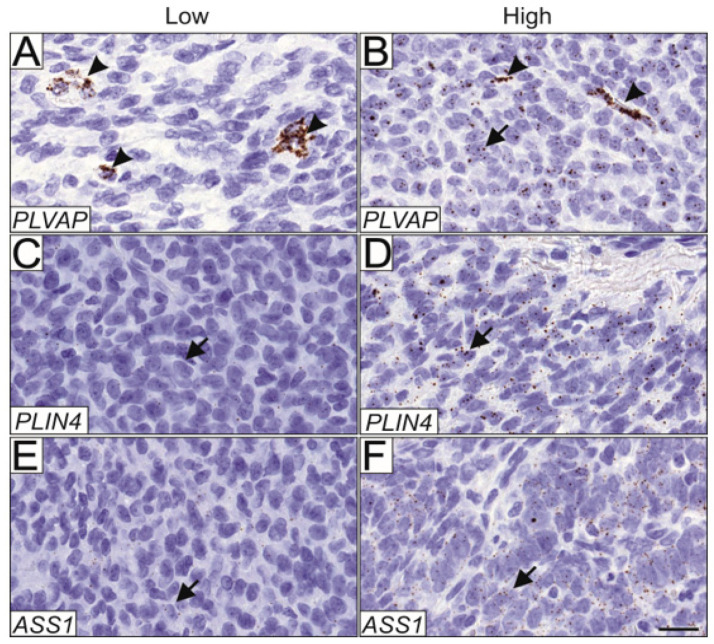
Validation of PLVAP, ASS1, and PLIN4 expression in AGCT tissue microarray. mRNA expression patterns of PLVAP, ASS1, and PLIN4 were assessed in a cohort of 175 AGCT samples using RNA in situ hybridization. Representative images of low and high expression patterns are shown. PLVAP was expressed in 33.8% of samples only in the endothelial cells (**A**), and both in endothelial and AGCT cells in 25.5% of the samples (**B**). Representative images of low and high ASS1 expression (**C**,**D**, respectively), and low and high PLIN4 expression (**E**,**F**, respectively) in AGCT cells. Magnification 164.5×, and scalebar 20 μm. Arrowheads indicate positively stained endothelial cells and arrows indicate positively stained AGCT cells.

*PLVAP* was expressed in both endothelial cells (Figure 4A) and tumor cells (Figure 4B), and expression in each cell type was scored separately. Compared to primary tumors, *PLVAP* expression was significantly higher in the tumor cells of all relapse samples (*p* = 0.02, *n* = 157). When primary tumors were correlated with only the first relapse the difference was even more significant (*p* = 0.0029, *n* = 143). Moreover, the endothelial cells of the relapsed tumors had significantly higher *PLVAP* expression compared to the endothelial cells of primary tumors (*p* < 0.0001, *n* = 156), and when only the first relapse was considered, the difference was more significant (*p* < 0.0001, *n* = 142). *PLVAP* expression was also higher in the endothelial cells of stage Ib-III primary tumors compared with endothelial cells of stage Ia primary tumors (*p* < 0.05, *n* = 105), but not in the tumor cells. *PLVAP* expression did not correlate with the growth pattern of the tumor, tumor size, or relapse. Matched pair analysis of primary and relapsed samples from the same patient (*n* = 17) did not reveal statistically significant differential expressions between the samples. In the primary tumors, *PLVAP* expression in either blood vessels or AGCT cells did not convey prognostic significance to either progression-free or overall survival.

*ASS1* and *PLIN4* mRNA were expressed primarily in the AGCT cells, although some signal was detected in stromal cells. Low and high expression patterns are shown for *ASS1* (Figure 4C,D, respectively) and *PLIN4* (Figure 4E,F, respectively). Using RNA in situ hybridization, we did not find statistically significant differences in *ASS1* and *PLIN4* expression between the primary and relapsed tumors in the TMA samples, nor did their expression correlate with primary tumor stage, size, growth pattern, or with relapse. Matched pair analysis of primary and relapsed samples from the same patients did not reveal statistically significant differential expression between the samples. Neither *PLIN4* nor *ASS1* expression in AGCT cells of the primary tumors conveyed prognostic significance to progression-free or overall survival.

## 4. Discussion

Due to its rarity, indolent growth pattern, and tendency to recur late, it is difficult to obtain cryopreserved high-quality AGCT samples with sufficient follow-up data to perform gene expression profiling for identification of markers that would predict tumor relapse. Archival formalin-fixed paraffin-embedded (FFPE) material collected at hospitals worldwide provide enormous resources for studying cancer and also enable long-term follow-up of the patients. FFPE tissue preservation is the most widely practiced method for archiving clinical samples, but the fixation causes substantial chemical modifications in the RNA, which makes the isolation of high-quality RNA challenging for genetic profiling. In addition, specimen size, tissue storage time and conditions influence the FFPE sample RNA quality [24]. For standard transcriptome level profiling, mRNA-sequencing of FFPE samples is, however, a reliable and cost-effective method. The gene expression measurement of FFPE samples using the standard poly(A) protocol represents similar differential expression as obtained for fresh frozen tissues [19]. In the present study, we used Lexogen QuantSeq 3′ mRNA-sequencing, which is suitable for detecting short transcripts of the partially degraded RNA of FFPE samples [25].

This is the first report to compare the gene expression profiles of primary AGCTs that did or did not recur in a group of patients with long follow-up. The cohort comprised of 35 archival *FOXL2*-mutation validated AGCT FFPE samples collected between 1975 and 2013 and sample archival age ranged from four to 43 years. We have comprehensive clinical data of these patients with exceptionally long follow-up times, ranging from 6.9 to 33.9 years, and this enabled us to perform retrospective comparisons between the non-relapsed and relapsed primary AGCT groups. AGCTs are generally non-inflamed or “cold” tumors that lack infiltrating T cells. The tumor mass consists mainly of tumor cells with minimal stroma [26]; therefore, our sequencing results are representative of AGCT cells. We performed mRNA sequencing on these samples, firstly, to compare the gene expression profiles of tumors with and without later relapse, and secondly, to compare the profiles of primary and relapsed tumors.

Interestingly, we found stable transcriptomes between primary tumors with or without relapse, suggesting that tumor relapse is not driven by alterations in gene expression profiles. This is consistent with the observed stable genomes of AGCTs [1,27]. In addition, analysis of the six matched pairs of primary and relapsed tumor showed no significant differentially expressed genes, further strengthening the role of stable transcriptomes during AGCT progression. Chromosomal alterations are relatively common in AGCT [12,13,28], and chromosome instability is suggested to predict early recurrence and aggressive tumor behavior [29]. The transcriptomic profile of the AGCT tumors have been shown to exhibit typical hallmarks of cancer [30]. In terms of AGCT prognosis, the significance of these findings remains unknown. The pathognomonic *FOXL2* missense mutation of AGCTs is not prognostic for relapse, albeit a study by Kraus et al. suggests that FOXL2 homozygous genotype is prevalent in recurrent AGCTs [29]. *FOXL2* target genes associated with faster cell cycling are induced, and genes linked with cell death are downregulated in AGCTs compared to healthy granulosa-luteal cells [30]. However, we did not observe differences in cell cycle associated genes between different AGCT groups. These findings highlight AGCT as a highly unique tumor, which lacks driver alterations in the canonical signaling pathways [31]. This lack of actionable driver alterations in the setting of relapse poses a therapeutic challenge and warrants further studies on the potential role of other factors, such as the tumor microenvironment and host immunology, in the prognostication and treatment of AGCTs.

Previously, next generation sequencing methods (NGS) have demonstrated a small number of recurring somatic mutations in AGCT that are enriched in relapsed tumors and may thus contribute to tumor progression. Pilsworth et al. discovered a C228T mutation in the *TERT* promoter of *FOXL2* validated AGCTs that is present in 23% of primary AGCTs and 45% of relapsed tumors [11,12,32]. A set of recurring truncating mutations in histone lysine methyltransferase (*KMT2D*/*MLL2*) gene was also found to be present in 3% of primary and in 23% of relapsed AGCTs studied [12]. Da Cruz et al. showed that AGCTs display intra-lesion heterogeneity and harbor both clonal and subclonal mutations [32]. However, the significance of these findings in AGCT tumor behavior remains unclear.

We found three significantly differentially expressed genes between the primary and relapsed tumors in the mRNA sequenced cohort; in relapsed tumors *PLVAP* was upregulated compared to primary tumors, while *ASS1* and *PLIN4* were downregulated. Further, *PLVAP* was significantly upregulated also in relapsed tumors in the validation TMA cohort. RNA in situ hybridization revealed that both AGCT cells and endothelial cells express *PLVAP,* and that it was upregulated in both cell types in relapsed tumors. PLVAP is an endothelial cell-specific protein that is upregulated in various pathophysiological processes associated with angiogenesis, including tumorigenesis [20]. It specifically localizes to diaphragms of fenestrae in fenestrated capillaries, and to stomatal diaphragms of caveolae [33]. This is the first report showing *PLVAP* expression in AGCT cells, but the function of this gene in the tumor cells remains unknown. Although *ASS1* and *PLIN4* expression were significantly downregulated in relapsed tumors in our RNA sequenced cohort, these findings were not recapitulated in the TMA cohort. The downregulation of *ASS1* and *PLIN4* is therefore unlikely to play a significant role in AGCT progression.

We acknowledge that the archival age of our samples affects their RNA quality and therefore reduces the power to detect differential gene expression. We, however, used a method successfully applied on archival samples in several recent studies to be able to study our unique cohort with extended follow-up times [34,35]. Therefore, we believe that our results are still valid and in line with previous findings; a subset of AGCT patients gain secondary mutations, but they do not explain the diverse clinical behavior of this disease, and the *FOXL2* C402G mutation remains the main driver of this disease [36]. The low number of differentially expressed genes between the primary and relapsed tumors suggests that AGCT do not exhibit major tumor evolution, at least in terms of global gene expression. As we measured only mRNA levels in this study, this study does not account for the role of post-translational editing and protein activity, or the role of microRNA expression profile in tumor progression. Furthermore, tumor progression may be partly explained by host immunology and immunoediting. AGCTs typically relapse late, and mutational burden accumulates slowly in the relapsed tumors [32]. It is tempting to speculate that *FOXL2* mutated tumor cells continue to proliferate slowly, so relapse may ensue if all residual tumor cells are not removed by surgery or if systemic immunoediting cannot control the tumor growth.

In summary, primary AGCTs that did or did not relapse show nearly identical gene expression patterns, and only *PLVAP* was found to be significantly differentially expressed between primary and relapsed tumors. Our results reinforce the previous findings that relapse and/or aggressive behavior of AGCT is not defined by activation or loss of specific genes or pathways. Instead, AGCT is a peculiar entity of low mutational burden, and we hypothesize that host-derived features, such as immunoediting, play an important role in the course of AGCT disease progression.

## Figures and Tables

**Table 1:** Clinical characteristics of the AGCT patients in the Lexogen QuantSeq sample cohort.

Characteristic	Primary Tumor Only	Primary Tumor with Recurrence	Recurrent	Total
	N (% or Range)	N (% or Range)	N (% or Range)	N (% or Range)
Number of patients	14	13	8	35
Growth pattern				
Sarcomatoid	5 (35.7)	8 (61.5)	4 (50)	17 (48.6)
Differentiated	9 (64.3)	5 (38.5)	4 (50)	18 (51.4)
Menopause status				
Pre-menopausal	5 (35.7)	4 (30.8)	1 (12.5)	10 (28.6)
Post-menopausal	9 (64.7)	9 (69.2)	7 (87.5)	25 (71.4)
Age at diagnosis in years (average, range)	52 (31–68)	48 (29–66)	57 (46–69)	51 (29–69)
Tumor size				
<10 cm	9 (64.7)	6 (46.2)	6 (75.0)	21 (60.0)
>10 cm	5 (35.7)	7 (53.8)	2 (25.0)	14 (40.0)
Time to 1st recurrence in years (average, range)	-	9.4 (1.8–18.9)	8.1 (2.8–18.9)	8.9 (1.8–18.9)
Follow-up time in years (average, range)	20.2 (17.2–26.1)	18.0 (6.9–33.9)	16.4 (7.4–22.2)	18.5 (6.9–33.9)
*TERT* promoter mutation status				
Positive	3 (21.4)	1 (7.7)	1 (12.5)	5 (14.3)
Wild type	10 (71.4)	7 (53.8)	1 (12.5)	18 (51.4)
N/A	1 (7.1)	5 (38.5)	6 (75.0)	12 (34.3)
Survival				
Alive	13 (92.9)	7 (53.8)	5 (62.5)	25 (71.4)
Died of AGCT	1 (7.1)	5 (38.5)	3 (37.5)	8 (22.9)
Died of other causes	1 (7.1)	1 (7.7)	-	2 (5.7)
Primary tumor stage				
1a	6 (42.9)	6 (46.2)	2 (25.0)	14 (40.0)
1c	5 (35.7)	6 (46.2)	5 (62.5)	16 (45.7)
2	2 (14.3)	1 (7.7)	1 (12.5)	4 (11.4)
3	1 (7.1)	-	-	
Sample archival age in years (average, range)	22 (18–27)	28 (13–43)	15 (4–35)	23 (4–43)

**Table 2:** Distribution of *PLVAP*, *PLIN4*, *ASS1*, and *PPIB* RNA in situ hybridization scores in AGCT TMA.

Marker	Score 0 *n*, (%)		Score 1 *n*, (%)		Score 2 *n*, (%)		Score 3 *n*, (%)	
*PLVAP* GCT cells (*n* = 168)	125	(74.4)	41	(24.4)	2	(1.2)	0	-
*PLVAP* endothelial cells (*n* = 168)	82	(48.8)	30	(17.9)	40	(23.8)	16	(9.5)
*PLIN4* (*n* = 168)	41	(24.4)	87	(51.8)	40	(23.8)	0	-
*ASS1* (*n* = 167)	69	(41.3)	88	(52.7)	10	(6.0)	0	-
*PPIB* (*n* = 168)	11	(6.5)	63	(37.5)	61	(36.6)	33	(19.6)

**Table 3:** Distribution of normalized *PLVAP*, *PLIN4*, and *ASS1* RNA in situ hybridization scores in AGCT TMA.

Marker	Positive *n*, (%)		Negative *n*, (%)	
*PLVAP* AGCT cells (*n* = 157)	43	(27.4)	114	(72.6)
*PLVAP* endothelial cells (*n* = 156)	84	(53.8)	72	(46.2)
*PLIN4* (*n* = 157)	126	(80.3)	31	(19.7)
*ASS1* (*n* = 157)	77	(49.4)	79	(50.6)

## Data Availability

The datasets generated and analyzed during the current study are available in the Gene Expression Omnibus (GEO) repository (https://www.ncbi.nlm.nih.gov/geo/, accessed on, accession number: GSE190942).
